# Supplementary material for: Modeling the initial phase of SARS-CoV-2 deposition in the respiratory tract mimicked by the 11C radionuclide
Source: PLoS One. 2021 Jan 14;16(1):e0245019. doi: 10.1371/journal.pone.0245019 (PMC7808619; doi:10.1371/journal.pone.0245019)

PLOS ONE

Modeling the initial phase of SARS-CoV-2 deposition in the respiratory tract mimicked by the 11C radionuclide

--Manuscript Draft--

**Manuscript Number:**	PONE-D-20-25540
**Article Type:**	Research Article
**Full Title:**	Modeling the initial phase of SARS-CoV-2 deposition in the respiratory tract mimicked by the 11C radionuclide
**Short Title:**	Lung deposition model for SARS-CoV-2
**Corresponding Author:**	Sérgio José Goncalves Jr., Ph.D.<br>Universidade do Estado do Rio de Janeiro<br>Rio de Janeiro, RJ BRAZIL
**Keywords:**	SARS-CoV-2; COVID-19; respiratory tract; viral load; radionuclides; ICRP-66
**Abstract:**	The knowledge on the deposition and retention of the viral particle of SARS-CoV-2 in the respiratory tract during the very initial intake from the ambient air is of prime importance to understand the infectious process and COVID-19 initial symptoms. To give some light on that, we propose to use a modified version of a widely tested lung deposition model developed by the ICRP, in the context of the ICRP Publication 66, that provides deposition patterns of microparticles in different lung compartments. In the model, we mimicked the "environmental decay" of the virus, determined by controlled experiments related to normal speeches, by the radionuclide 11 C that presents comparable decay rates. Our results confirm clinical observations on the high virus retentions observed in the extrathoracic region and the lesser fraction on the alveolar section (in the order of 5), which relevance is a subject to be investigated.
**Order of Authors:**	Heitor Evangelista
	César Amaral
	Luís Cristóvão Porto
	Sérgio J. Goncalves Jr., Ph.D.
	Eduardo Delfino Sodré
	Juliana Nogueira
	Angela M.G. dos Santos
	Marcio Cataldo
**Additional Information:**	
**Question**	**Response**
**Financial Disclosure**<br><br>Enter a financial disclosure statement that describes the sources of funding for the work included in this submission. Review the submission guidelines for detailed requirements. View published research articles from *PLOS ONE* for specific examples.<br><br>This statement is required for submission and **will appear in the published article** if the submission is accepted. Please make sure it is accurate.	The authors received no funding for this research.The funders had no role in study design, data collection and analysis, decision to publish, or preparation of the manuscript.

Powered by Editorial Manager® and ProduXion Manager® from Aries Systems Corporation

**Unfunded studies**<br>Enter: *The author(s) received no specific funding for this work.*<br><br>**Funded studies**<br>Enter a statement with the following details:<br>• Initials of the authors who received each award<br>• Grant numbers awarded to each author<br>• The full name of each funder<br>• URL of each funder website<br>• Did the sponsors or funders play any role in the study design, data collection and analysis, decision to publish, or preparation of the manuscript?<br>• **NO** - Include this sentence at the end of your statement: *The funders had no role in study design, data collection and analysis, decision to publish, or preparation of the manuscript.*<br>• **YES** - Specify the role(s) played.<br><br>* typeset	
**Competing Interests**<br><br>Use the instructions below to enter a competing interest statement for this submission. On behalf of all authors, disclose any competing interests that could be perceived to bias this work—acknowledging all financial support and any other relevant financial or non-financial competing interests.<br><br>This statement **will appear in the published article** if the submission is accepted. Please make sure it is accurate. View published research articles from *PLOS ONE* for specific examples.	The authors have declared that no competing interests exist.

**NO authors have competing interests**<br><br>Enter: *The authors have declared that no competing interests exist.*<br><br>**Authors with competing interests**<br><br>Enter competing interest details beginning with this statement:<br><br>*I have read the journal's policy and the authors of this manuscript have the following competing interests: [insert competing interests here]*<br><br>* typeset	
**Ethics Statement**<br><br>Enter an ethics statement for this submission. This statement is required if the study involved:<br><br>• Human participants<br>• Human specimens or tissue<br>• Vertebrate animals or cephalopods<br>• Vertebrate embryos or tissues<br>• Field research<br><br>Write "N/A" if the submission does not require an ethics statement.<br><br>General guidance is provided below. Consult the submission guidelines for detailed instructions. **Make sure that all information entered here is included in the Methods section of the manuscript.**	The use of COVID-19 data was approved by the Ethics in Research Committee of the Pedro Ernesto University Hospital under the project “Epidemiological, laboratorial, and clinical profile of the COVID-19 pandemic of patients treated at Rio de Janeiro State University (UERJ)” (CAAE: 30135320.0.0000.5259). All COVID-19 typing data were de-identified prior to the analysis and available for the authors uniquely as totals, as presented on Table 2.

**Format for specific study types**<br><br>**Human Subject Research (involving human participants and/or tissue)**<br>• Give the name of the institutional review board or ethics committee that approved the study<br>• Include the approval number and/or a statement indicating approval of this research<br>• Indicate the form of consent obtained (written/oral) or the reason that consent was not obtained (e.g. the data were analyzed anonymously)<br><br>**Animal Research (involving vertebrate animals, embryos or tissues)**<br>• Provide the name of the Institutional Animal Care and Use Committee (IACUC) or other relevant ethics board that reviewed the study protocol, and indicate whether they approved this research or granted a formal waiver of ethical approval<br>• Include an approval number if one was obtained<br>• If the study involved *non-human primates*, add *additional details* about animal welfare and steps taken to ameliorate suffering<br>• If anesthesia, euthanasia, or any kind of animal sacrifice is part of the study, include briefly which substances and/or methods were applied<br><br>**Field Research**<br><br>Include the following details if this study involves the collection of plant, animal, or other materials from a natural setting:<br>• Field permit number<br>• Name of the institution or relevant body that granted permission	
**Data Availability**<br><br>Authors are required to make all data underlying the findings described fully available, without restriction, and from the time of publication. PLOS allows rare exceptions to address legal and ethical concerns. See the PLOS Data Policy and FAQ for detailed information.	Yes - all data are fully available without restriction

A Data Availability Statement describing where the data can be found is required at submission. Your answers to this question constitute the Data Availability Statement and **will be published in the article**, if accepted.<br><br>**Important:** Stating 'data available on request from the author' is not sufficient. If your data are only available upon request, select 'No' for the first question and explain your exceptional situation in the text box.<br><br>Do the authors confirm that all data underlying the findings described in their manuscript are fully available without restriction?	
**Describe where the data may be found in full sentences. If you are copying our sample text, replace any instances of XXX with the appropriate details.**<br><br>• If the data are **held or will be held in a public repository**, include URLs, accession numbers or DOIs. If this information will only be available after acceptance, indicate this by ticking the box below. For example: *All XXX files are available from the XXX database (accession number(s) XXX, XXX.).*<br>• If the data are all contained **within the manuscript and/or Supporting Information files**, enter the following: *All relevant data are within the manuscript and its Supporting Information files.*<br>• If neither of these applies but you are able to provide **details of access elsewhere**, with or without limitations, please do so. For example:<br><br>*Data cannot be shared publicly because of [XXX]. Data are available from the XXX Institutional Data Access / Ethics Committee (contact via XXX) for researchers who meet the criteria for access to confidential data.*<br><br>*The data underlying the results presented in the study are available from (include the name of the third party*	All relevant data are within the manuscript and its Supporting Information files.

and contact information or URL).<br>• This text is appropriate if the data are owned by a third party and authors do not have permission to share the data.<br><br>* typeset	
Additional data availability information:	

Powered by Editorial Manager® and ProduXion Manager® from Aries Systems Corporation

Manuscript

Click here to access/download;Manuscript;MODELING THE INITIAL PHASE OF SARS _evangelista et al 2020.docx

MODELING THE INITIAL PHASE OF SARS-COV-2 DEPOSITION IN THE RESPIRATORY TRACT MIMICKED BY THE ^{11}C RADIONUCLIDE

Heitor Evangelista[1], Cesar Amaral[1,4], Luís Cristóvão Porto[2], Sergio J. Gonçalves Junior[1], Eduardo Delfino Sodré[1], Juliana Nogueira[1], Angela M.G. dos Santos[2], Marcio Cataldo[3]

(1)LARAMG - Laboratory of Radioecology and Global Changes/DBB/IBRAG. Uerj, Pavilhão Haroldo L. Cunha, Rua São Francisco Xavier 524. Maracanã, Rio de Janeiro State University, Rio de Janeiro, RJ, 20550-013. Brazil.

(2)HLA - Laboratório de Histocompatibilidade e Criopreservação, Rio de Janeiro State University. Av. Mal. Rondon, 381 - São Francisco Xavier, Rio de Janeiro, RJ, 20950-003. Brazil.

(3)Draxos Consultoria e Gestão Ambiental Ltda. Av. das Américas 7938 - Sala 329, Rio de Janeiro, RJ, 22793-081. Brazil.

(4)LDD – Laboratorio de Diagnósticos por DNA/DECOL/IBRAG. Uerj, Pavilhão Haroldo L. Cunha, Rua São Francisco Xavier 524. Maracanã, Rio de Janeiro State University, Rio de Janeiro, RJ, 20550-013. Brazil.

Keywords: SARS-CoV-2, COVID-19, respiratory tract, viral load, radionuclides, ICRP-66

Abstract

The knowledge on the deposition and retention of the viral particle of SARS-CoV-2 in the respiratory tract during the very initial intake from the ambient air is of prime importance to understand the infectious process and COVID-19 initial symptoms. To ~~give some light on~~ that, we propose to use a modified version of a widely tested lung deposition model developed by the ICRP, in the context of the ICRP Publication 66, that provides deposition patterns of microparticles in different lung compartments. In the model, we mimicked the "environmental decay" of the virus, determined by controlled experiments related to normal speeches, by the radionuclide ^{11}C that presents comparable decay rates. Our results confirm clinical observations on the high virus retentions observed in the extrathoracic region and the lesser fraction on the alveolar section (in the order of 5), which relevance is a subject to be investigated.

Introduction

Respiratory infections are relevant clinical conditions due to their diffusion, and potentially severe consequences, such as presently observed for the SARS-CoV spread all around the world. Declared as a "global pandemic" by the World Health Organization on March 11th, 2020, the new coronavirus disease 2019 (COVID-19) represents one of the most significant public health challenges in recent decades [1]. Respiratory infections caused by viruses, mainly due to their high capacity of infection and spread, are a major cause of illness and death from a worldwide perspective. In 2003, with the

global alert caused by the SARS (Severe Acute Respiratory Syndrome), the coronaviruses joined this group, triggered by a newly identified coronavirus (SARS-CoV). Since then, several other pathogens associated with acute respiratory system disorders have been described, such as more aggressive strains of Influenza, MERS-CoV in the Middle East, and also new types of coronavirus NL63 and KHU1 [2-3]. Therefore, recognizing the modes of transmission of these emerging infectious diseases is a vital factor for both the safety of health staff, who interact with infected individuals, and the public, who will be exposed sooner or later to areas where these agents are circulating.

Airborne transmission is a key issue for the understanding of SARS-CoV-2 spread out, and it is particularly important for the community of healthcare professionals since they are more exposed to infected patients. However, the potential through contagion for the overall population related to the high agglomeration of citizens, as observed in various modes of urban transport in large cities, especially in developing countries, is not yet fully clarified. In Italy (Bergamo city), a first study showed that RNA SARS-CoV-2 might be present in association with microparticles in the outdoor environment. Still, the detection of the virus itself was mostly inconclusive [4]. While in the free atmosphere, virus particles undergo natural denaturation or inactivation conditions due to solar radiation, relative humidity, and air temperature [5-6]. This condition leads to the dehydration of virus particles caused by speech, sneezing, or coughing. As a result, the virus particles agglutinate with other organic and inorganic molecules/particles suspended in the air, and this interaction causes their size distribution to change. Therefore, this will affect its diffusion/dispersion/deposition and residence time in the atmosphere. Unlike other stochastic models (that is, the fraction of deposition in the lung compartment is determined based on the aerodynamic properties of aerosols), we here provide a simulation of a model that takes into account the full biokinetics of the radionuclide ^{11}C mimicking particles containing viruses. To perform that, we used a modified version of a widely tested lung deposition model developed by the International Commission on Radiological Protection (ICRP), in the context of the ICRP Publication 66 (Human Respiratory Tract Model for Radiological Protection-66) from the ICRP Task Group on Internal Dosimetry.

Materials and Methods

During the last decades, several studies have deeply investigated the dynamics of respiratory infections to gain information on effective treatment/prevention of these clinical events [7]. Many different models have been developed till then. A respiratory infection model is a system that emulates the complexities observed on the relationship between the infectious agents and the host's defenses. Several *in vivo* and *in vitro* respiratory models for humans and other vertebrates are available and could be easily reproduced. Mathematical models are also available and were created to numerically describe the principles and evolution of respiratory infections and their diffusion. All of them require specific inputs and have complexities related to the condition they attempt to emulate, therefore presenting both advantages and limitations.

The ICRP Model

The ICRP has developed models for aerosol pulmonary deposition, given the intensive use of natural and artificial radionuclides in the nuclear industry. Such activities range from uranium mining, where workers are exposed to dust containing naturally occurring radioactive aerosols [8], to sectors where there is the handling of wastes from nuclear power plants and related facilities [9-10]. Such models depend on the aerosol size distribution of the radioactive aerosols suspended in the air, their chemical form, and its solubility and the corresponding biokinetic processes associated [11]. In this context, extensive research on the biokinetics of radioactive aerosols has led to successful practical application

and has been greatly improved in calculating the internal dose of workers and people, especially those living in regions with high levels of natural radioactivity [12].

Herein we use a radionuclide-based model starting with the premise that the virus (SARS-Cov-2) has an "environmental half-life" due to its degradation process in the environment before the deposition in the respiratory tract. From this assumption, we draw an analogy with the half-life of a radionuclide. Thus, we can attribute to the virus an analog "constant of disintegration" related to the outdoor environment. The choice of the radionuclide was made based on studies by Stadnytskyi et al. [13], which used a highly sensitive laser light scattering system to track the dynamics of airborne speech droplets generated by carriers with SARS-CoV-2. Droplets were produced during the normal speaking condition, and can be suspended in the air for a tenth of minutes and, therefore, can be a potential source of airborne virus transmission in low disturbed places. The settling down (vanishing) time-scale of droplets before dehydration and attachment to other existing microparticles in the atmosphere describes a behavior similar to the artificial radioisotope ^{11}C disintegration curve, which is 20.33 minutes in half-life (Fig.1a). ^{11}C is one of the most useful radionuclides employed for Positron Emission Tomography (PET) radiochemistry because its attachment to a biologically active molecule does not modify the biochemical properties of the inoculated compound [14]. From the above, we elected the ^{11}C to mimic the virus intake and deposition in the respiratory tract. Another reason relies on the fact that carbon molecules are one of the base constituents of cells and tissues; this allows very precise information on metabolism processes, receptor/enzyme function, and biochemical mechanisms. As a complementary input to the model, we used airborne SARS-CoV-2 size distribution data, by Liu et al. [15], in a stand of theoretical curves or data derived from other virus types as influenza, Fig. 1b. On that study, the size distribution of viral particles from a sequence of speeches in pre-sterilized gelatin filters installed inside a miniature cascade impactor during an air monitoring in the Renmin Hospital of Wuhan University - China, in February-March 2020 was obtained. As a result, they found that the aerodynamical diameter of particles varied from 5.0 to 5.5 μm. Other studies of droplets produced during speeches with sustained vocalization found modes from 1.8 to 5.5 μm [16].

Fig. 1 Basic inputs for the lung deposition model: (a) disintegration curve of the radionuclide ^{11}C (in bold) and speech droplet nuclei vanishing (dotted line) and variance (gray shaded area); (b) size distribution of SARS-Cov-2 obtained by Liu et al., (2020) [15]; (c) respiratory tract compartments investigated by the model: AI (alveolar), bb (bronchioles), BB (bronchi), ET_1 (extrathoracic region 1 - retention of material deposited in the anterior nose) and ET_2 (extrathoracic region 2 – retention of material deposited in the posterior nasal passage, larynx, pharynx, and mouth).

The imperative assumption to allow the use of the ICRP model, which is specially designed for use by radionuclides, is to attribute a "decay property" to the virus. However, since the virus is no longer subject to any "decay" after it has been absorbed by the human organism, the assumption of mimicry by ^{11}C stops at that stage. The concentration of active virus particles inoculated in the body remains the same until physiological reactions begin, and virus elimination/cell infections and replication occur. It, therefore, means that our estimates refer only to the initial deposition of the virus and that the deposition fractions presented here have been calculated for the first 1 minute after deposition.

The model used subdivides the respiratory system into two basic compartments: the extrathoracic region (composed of nose and trachea) and the thoracic region (composed of the bronchi to the alveolar sacs). The compartments AI, bb, BB, ET_1, and ET_2 represent the alveolar, bronchioles, bronchi, and extrathoracic regions, respectively, Fig. 1c. The lung is considered to have 16 generations where bifurcations occur in the bronchial and bronchioles tree. The BB region comprises the generations from 0 to 8 and the bb from 9 to 15. The rates of mechanical removal of a compartment are expressed in

1/time. Ciliary transport and retention time in the tissues migrating to the pulmonary lymph nodes are also considered in the model, as well as blood absorption from these compartments, causing then a dispute between mechanical removal and blood absorption. The compartments above act as source compartments in the infection of the lung tissues. All the calculations were performed by the AIDE (Activity and Internal Dose Estimates) software [17]. The basic inputs of the model are presented in Table 1, where AMAD means "aerosol containing the viral load" median aerodynamic diameter, which implies that 50% of the viral load in the aerosol is associated with particles of aerodynamic diameter greater than the AMAD (used when deposition depends mainly on inertial impaction and sedimentation). At the same time, a compound refers to a material classified according to its rate of absorption from the respiratory tract to body fluids. In this case, deposited Type F materials are those that are readily absorbed into body fluids from the respiratory tract (Fast absorption), and f_1 is the fractional absorption in the gastrointestinal tract.

Table 1. Specifications of model parameters and inputs.

AIDE model parameters	AIDE model inputs
**Model**	C, Isotope: C-11
**Data source**	Carbon according to ICRP-67*
**Subject**	Reference Worker (breathing rate of 1.2 m^3 h^{-1})
**Intake type**	Inhalation, Single
**Initial or Daily Activity**	1.000E+00 Bq
**Inhalation type**	Respiratory Tract model: ICRP-66
**Compound**	Type F
**GI Tract Absorption Factor**	f_1 = 1.000E+00
**AMAD (μm)**	5.000E+00**

* ICRP, 1993. Age-dependent Doses to Members of the Public from Intake of Radionuclides – Part 2 Ingestion Dose Coefficients. ICRP Publication 67. Ann. ICRP 23 (3-4)

** Based on the geometric mean of Fig.1b.

SARS-Cov-2 Field Data

Considering the fact that COVID-19 is highly associated through the respiratory airways, we also present data of nasopharyngeal swabs (1490) and bronco-tracheal aspirate (4) COVID-19 RT-PCR tests performed at Pedro Ernesto University Hospital (HUPE), one of the specialized units for COVID-19 treatment in the Rio de Janeiro City/Brazil. We used the data to observe the evolution of the pandemics moments just before and after the mandatory use of personal safety equipment (masks) has been implemented by the local government. The database for patients with COVID-19 started on March 29th (13th epidemiological week) up to July 12th (28th epidemiological week), when 939 patients were hospitalized, and a total of 1,494 individuals were tested at the HUPE. Among the realized tests, all the four bronco-tracheal aspirates resulted in negative for SARS-CoV-2. The remaining test results were presented in Table 2.

Table 2. The number of detected new and total tests at Pedro Ernesto University Hospital from Epidemiological week 13 to 28.

		Hospitalizations			Total		
**Epidemiological Week**	**Date**	**Tests**	**Detected**	**(%)**	**Tests**	**Detected**	**(%)**
13	29-mar	11	2	18.2	11	2	18.2
14	5-abr	22	7	31.8	24	8	33.3
15	12-abr	40	16	40.0	48	19	39.6
16	19-abr	46	40	87.0	58	50	86.2
17	26-abr	42	36	85.7	47	40	85.1
18	3-mai	86	52	60.5	112	63	56.3
19	10-mai	93	55	59.1	142	74	52.1
20	17-mai	56	31	55.4	108	57	52.8
21	24-mai	53	27	50.9	91	35	38.5
22	31-mai	106	46	43.4	179	74	41.3
23	7-jun	74	21	28.4	117	29	24.8
24	14-jun	72	19	26.4	119	30	25.2
25	21-jun	54	8	14.8	117	17	14.5
26	28-jun	59	10	16.9	126	26	20.6
27	5-jul	55	4	7.3	91	7	7.7
28	12-jul	70	5	7.1	104	9	8.7

Results

Our results point to a far more relevant deposition fraction at the extrathoracic compartments of ET1 accounting to 47.4 and ET2 to 48.78 for the virus intake after 1 minute. In contrast, the sum for the bronchial and bronchioles compartments corresponded to 3.75 (Fig. 2).

Fig 2. Time-dependent particle transport from each respiratory tract region in the compartments of the model for SARS-Cov-2 (after 1 minute of exposure): (a) and (b) time response curves; (c) and (d) fractions of the initial intake of the virus by ET_1 pathway.

Discussions

For a comparison with a Stochastic Lung Deposition Model, as proposed by Madas et al. [18], they found 61.8% of the total inhaled mass fraction to be at the upper airways, ~8.5% for the acinar airways and ~5.5% for the bronchial compartment corresponding to a single inhalation. The differences between the two models can be attributed to the fact that Madas et al. [18] have used a mass size distribution of particles from influenza with modal values between 2-3 µm, derived from coughing of patients, while we used a SARS-Cov-2 data in conditions of speeches with AMAD ~ 5 µm. Our results stress the impact of the upper airways in the initial airborne virus retention in the respiratory tract since the total extrathoracic compartment may retain more than 96% of the virus load. Both models point to at least ~4% of contribution to the most inner parts of the lung (bronchial and bronchioles). In the SARS-Cov-2 size distribution we have used, it is evident an existing viral load in the ultrafine particle size range (< 0.1 mm diameter). Their behavior differs from other groups of particles as the fine and coarse modes, since the virus attached to ultrafine particles may be deposit in the inner lung

compartments by diffusion mechanisms. From controlled experiments, it is known that ultrafine particles peak deposition occurs in lung regions that encompass the transition zone between the conducting airways and the alveolar region [19]. Therefore, as our model predicts a small fraction of virus-containing particles reaching directly to the alveolar region, its significance in disease development should be considered. The SARS-Cov-2 can bind to the cells in that compartment via ACE2 (Angiotensin-Converting Enzyme 2), which are the host cell receptor responsible for mediating SARS-CoV-2 infection [20]. ACE2 plays an important role in the lungs protecting it from acute respiratory distress syndrome (ARDS) by breaking down Angiotensin II, which has inflammatory effects [21-22]. Binding of the SARS-CoV-2 to ACE2 inhibits it and thus weakens its protective action on the organ [23].

The pathogenesis and virus transmission pathways are still being investigated, and the already published results are, in fact, even under intense debate. That is the case for the airborne transmission of the virus. The analyses of the temporal dynamics of the SARS-Cov-2 infection indicates that the viral shedding begins after 2-3 days of the first symptoms, thereby promoting pre-symptomatic/asymptomatic transmission of the virus [24]. In this sense, understanding the first phases of the virus infection is paramount. Our results were derived from normal speech conditions as viral source-term and indoor environments. In this specific case, we observed extrathoracic percentage infection levels in good agreement with clinical observations of patients that initially presented mild COVID-19 symptoms evolving to a more deteriorated health board [25]. Though COVID-19 manifestation linked to a minimal infectious dosage, as expected in the alveolar compartment, is not yet clear, given that: (1) no masks have a 100 % retention efficiency; (2) SARS-CoV-2 viral particles are found in aerodynamic diameters shorter than 0.1 μm in surveys; (3) the model predicts around 5% direct penetration of viruses in the alveolar compartments. These facts, when combined, may explain why a fraction of the population, even using masks, get contaminated with "no apparent reason." Respiratory tract models developed by the ICRP Task Groups have been largely used for safety and protection in nuclear activities in several countries, reaching excellent performance and being validated by internal measurements. Their use for non-radioactive aerosols of biological and mineral origin and pollutants is an emerging topic and a potential to be explored.

Since ET1 and ET2 are key-compartments in the initial phase of SARS-Cov-2 deposition, we investigated the impact of the use of individual protection such as masks and face shields on the epidemiological data related to the COVID-19. Table 2 shows a survey developed by our working group from nasopharyngeal swabs and bronco-tracheal aspirate tests for COVID-19 by RT-PCR performed at Pedro Ernesto University Hospital (HUPE), in Rio de Janeiro, Brazil. The city of Rio de Janeiro in no time had adopted a full lockdown strategy, but just recommendations of social distance and personal care such as hands washing and alcohol use for individual and items/objects disinfection. However, COVID-19 data showed continuous prevalence forcing the local authorities to carry out a mandatory use of masks on April 23rd.

Our results suggest that the fraction of both hospitalized and total tested patients with SARS-CoV-2 detected from the RT-PCR tests exhibited a significant decrease when we observed an immediate drop in percentage from 85% to 60% (Figure 3). We do believe that the analyzed sample is representative of the city population since the HUPE receives patients from several health units spread all over the urban domain. We should also note that due to socio-economic reasons and also difficulties in the acquisition of high-quality safety equipment, most of the population made use of simple, cheap, and home-made masks and face shields. However, despite these circumstances, effective response against the pandemic was achieved.

Fig. 3 SARS-CoV-2 detection from nasopharynx COVID-19 RT-PCR tests performed at Pedro Ernesto University Hospital (HUPE) in Rio de Janeiro/Brazil.

In summary, the respiratory tract models produced by IAEA Task Group have been widely used in many countries for health and defense in nuclear activities, reaching excellent performance and being validated by *in locus* measurements. Their use for non-radioactive aerosols and microparticles of biological and mineral origin and pollutants is an emerging topic and a potential to be explored.

Conflict of Interest

The authors declare that the research was conducted in the absence of any commercial or financial frameworks that could be construed as a potential conflict of interest.

Funding

The authors received no funding for this research.

Acknowledgments

We greatly thank Dr. Luiz Bertelli for the availability of AIDE (Activity and Internal Dose Estimates) software and important comments on the numeric model and Roberta Priori for draw designs.

References

1. Santarpia JL, Rivera DN, Herrera V, Morwitzer MJ, Creager H, Santarpia GW, et al. Aerosol and Surface Transmission Potential of SARS-CoV-2. medRxiv. 2020:2020.03.23.20039446.

2. Van Der Hoek L, Pyrc K, Jebbink MF, Vermeulen-Oost W, Berkhout RJ, Wolthers KC, et al. Identification of a new human coronavirus. *Nature medicine*. 2004;10(4):368-73. doi: 10.1038/nm1024

3. Woo PC, Lau SK, Chu C-m, Chan K-h, Tsoi H-w, Huang Y, et al. Characterization and complete genome sequence of a novel coronavirus, coronavirus HKU1, from patients with pneumonia. *Journal of virology*. 2005;79(2):884-95. doi: 10.1128/JVI.79.2.884-895.2005

4. Setti, L., Passarini, F., De Gennaro, G., Barbieri, P., Perrone, M.G., Borelli, M., Palmisani, J., Di Gilio, A., Torboli, V., Pallavicini, A., Ruscio, M., Piscitelli, P., Miani, A. SARS-Cov-2 RNA Found on Particulate Matter of Bergamo in Northern Italy: First Preliminary Evidence *medRxiv preprint* . 2020; doi: 10.1101/2020.04.15.20065995

5. Chan, K. H., Peiris, J. S. M., Lam, S. Y., Poon, L. L. M., Yuen, K. Y., & Seto, W. H. (2011). The effects of temperature and relative humidity on the viability of the SARS coronavirus. *Advances in Virology*. doi: 10.1155/2011/734690

6. Shanna Ratnesar-Shumate, Gregory Williams, Brian Green, Melissa Krause, Brian Holland, Stewart Wood, Jordan Bohannon, Jeremy Boydston, Denise Freeburger, Idris Hooper, Katie Beck, John Yeager, Louis A Altamura, Jennifer Biryukov, Jason Yolitz, Michael Schuit, Victoria Wahl, Michael Hevey, Paul Dabisch, Simulated Sunlight Rapidly Inactivates SARS-CoV-2 on Surfaces, *The Journal of Infectious Diseases*, Volume 222, Issue 2, July 15th 2020, Pages 214–222, https://doi.org/10.1093/infdis/jiaa274

7. Saturni S, Contoli M, Spanevello A, Papi A. Models of Respiratory Infections: Virus-Induced Asthma Exacerbations and Beyond. *Allergy, asthma & immunology research*. 2015;7:525–533. doi: 10.4168/aair.2015.7.6.525.

8. Otahal P, Burian I. The airborne natural radioactivity in the uranium mine Rožná I. *Radiation protection dosimetry*. 2011;145(2-3):150-4.doi: 10.1093/rpd/ncr046

9. Breustedt B, Giussani A, Noßke D. Internal dose assessments–Concepts, models and uncertainties. *Radiation Measurements*. 2018;115:49-54. doi: 10.1016/j.radmeas.2018.06.013

10. Oberdörster G. Airborne cadmium and carcinogenesis of the respiratory tract. *Scandinavian journal of work, environment & health.* 1986:523-37. doi: 10.5271/sjweh.2104

11. Cuddihy R, McClellan R, Griffith W. Variability in target organ deposition among individuals exposed to toxic substances. *Toxicology and applied pharmacology*. 1979;49(2):179-87. doi: 10.1016/0041-008X(79)90240-0

12. Henricsson F, Persson BR. Polonium-210 in the biosphere: biokinetics and biological effects. In Radionuclides: sources, properties and hazards. *Nova Science*, Hauppauge. 2012.

13. Stadnytskyi V, Bax CE, Bax A, Anfinrud P. The airborne lifetime of small speech droplets and their potential importance in SARS-CoV-2 transmission. *Proceedings of the National Academy of Sciences*. 2020;117(22):11875-7.doi: 10.1073/pnas.2006874117

14. Yang L, Scott PLH, Shao X. [11-C] Carbon Dioxide: Starting Point for Labeling PET Radiopharmaceuticals. *Carbon Dioxide Chemistry, Capture and Oil Recovery*. Chapter 7. 2018; 123-138. doi:10.5772/intechopen.72313

15. Liu Y, Ning Z, Chen Y, Guo M, Liu Y, Gali NK, et al. Aerodynamic analysis of SARS-CoV-2 in two Wuhan hospitals. *Nature*. 2020;582(7813):557-60. doi: 10.1038/s41586-020-2271-3

16. Morawska L, Johnson G, Ristovski Z, Hargreaves M, Mengersen K, Corbett S, et al. Size distribution and sites of origin of droplets expelled from the human respiratory tract during expiratory activities. *Journal of Aerosol Science*. 2009;40(3):256-69.

17. Bertelli L, Melo D, Lipsztein J, Cruz-Suarez R. AIDE: internal dosimetry software. *Radiation Protection Dosimetry.* 2008;130(3):358-67. doi: 10.1093/rpd/ncn059

18. Madas BG, Füri P, Farkas A, Nagy A, Czitrovszky A, Balásházy I, Schay GG, Horváth A. Deposition distribution of the new coronavirus (SARS-CoV-2) in the human airways upon exposure to cough-generated aerossol. 2020. *medRxiv*; doi: 10.1101/2020.05.13.20100057

19. Kim CS, Jaques PA. Respiratory dose of inhaled ultrafine particles in healthy adults. *Philosophical Transactions of the Royal Society of London Series A: Mathematical, Physical and Engineering Sciences.* 2000;358(1775):2693-705. doi: 10.1098/rsta.2000.0678

20. Li WH, Moore MJ, Vasilieva NY, et al. Angiotensin-converting enzyme 2 is a functional receptor for the SARS coronavirus. *Nature*. 2003; 426: 450–454. doi: 10.1038/nature02145

21. Imai Y, Kuba K, Penninger JM. (2008). The discovery of angiotensin-converting enzyme 2 and its role in acute lung injury in mice. *Experimental Physiology*, 2008. *93(5)*, *543–548.* doi:10.1113/expphysiol.2007.040048

22. Jia H. Pulmonary Angiotensin-Converting Enzyme 2 (ACE2) and Inflammatory Lung Disease. *Shock*. Augusta, Ga.2016. 46(3): 239-48. doi:10.1097/SHK.0000000000000633. PMID 27082314

23. Kuba K, Imai Y, Rao S *et al.* A crucial role of angiotensin converting enzyme 2 (ACE2) in SARS coronavirus–induced lung injury. *Nat Med.* 2005. *11*. 875–879 (2005). doi:10.1038/nm1267

24. He X, Lau EHY, Wu P, Deng X, Wang J, Hao X, et al. Temporal dynamics in viral shedding and transmissibility of COVID-19. *Nature Medicine*. 2020;26(5):672-5. doi: 10.1038/s41591-020-0869-5

25. Wu C, Chen X, Cai Y, Xia Ja, Zhou X, Xu S, et al. Risk Factors Associated With Acute Respiratory Distress Syndrome and Death in Patients With Coronavirus Disease 2019 Pneumonia in Wuhan, China. *JAMA Internal Medicine*. 2020;180(7):934-43. doi: 10.1001/jamainternmed.2020.0994

Author Contributions

All authors contributed equally to the development and text of the manuscript. Heitor Evangelista (Project coordinator, creator of the project), Cesar Amaral (project co-author, participation in the text content and discussions) Luís Cristóvão Porto (medical survey), Sergio Gonçalves Junior (participation in the text content and discussions), Eduardo Delfino Sodré (participation in the text content and discussions), Juliana Nogueira (numeric model analysis, Marcio Cataldo Gomes da Silva (participation in the text content and discussions).

Figure 1

Click here to access/download;Figure;Fig1.tif

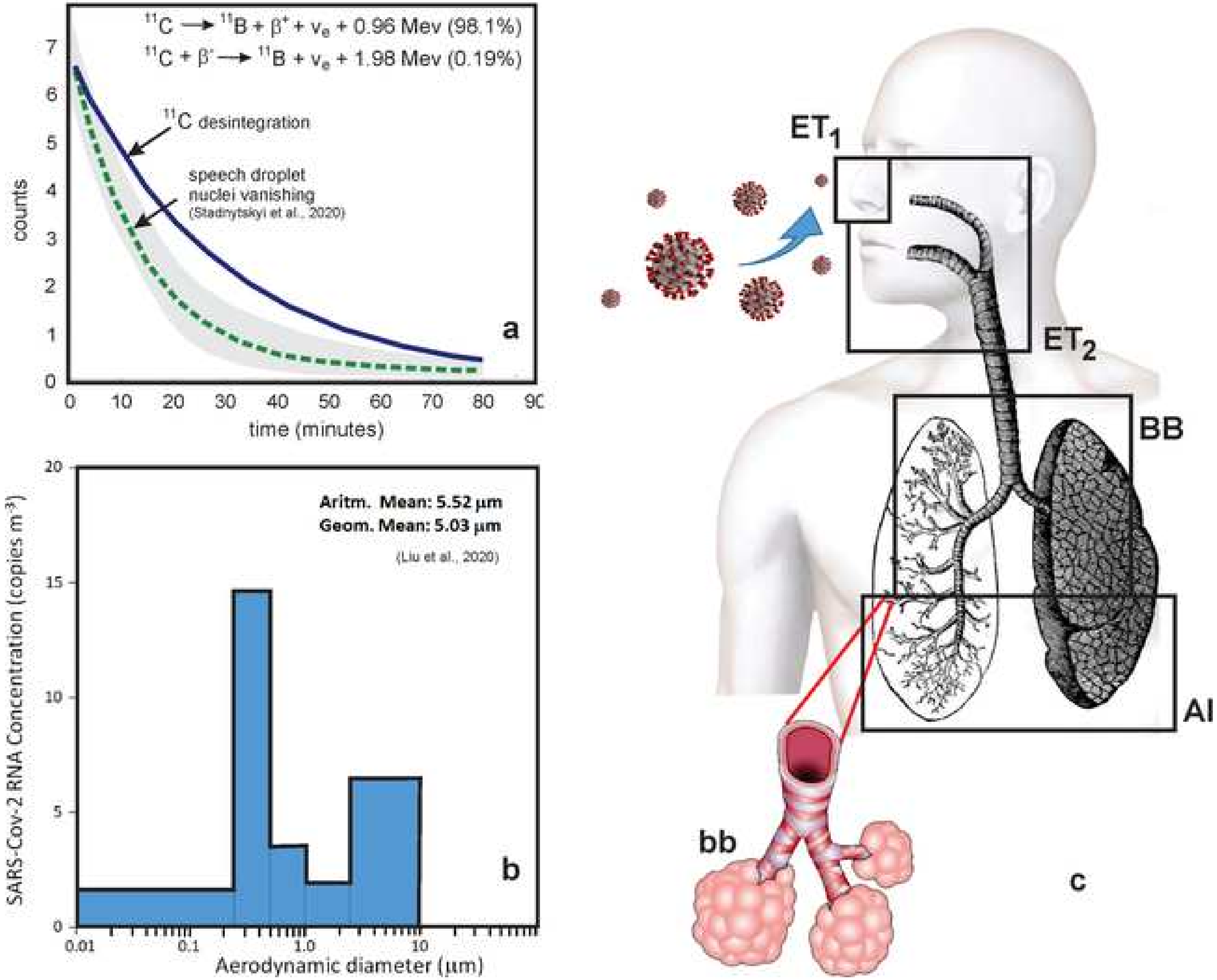

Figura 2

Click here to access/download;Figure;Fig2.tif

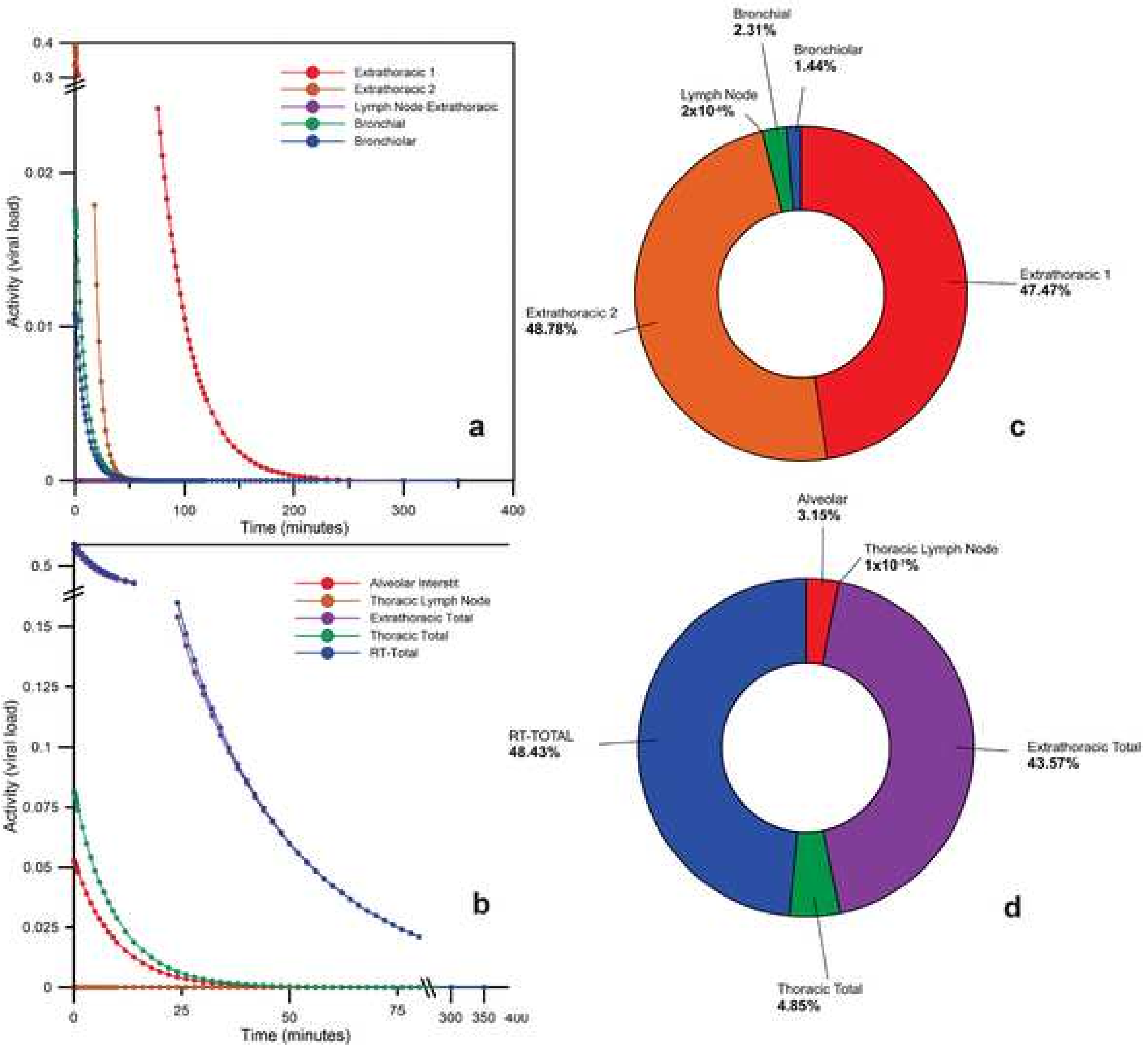

Figure 3

Click here to access/download;Figure;Fig3.tif

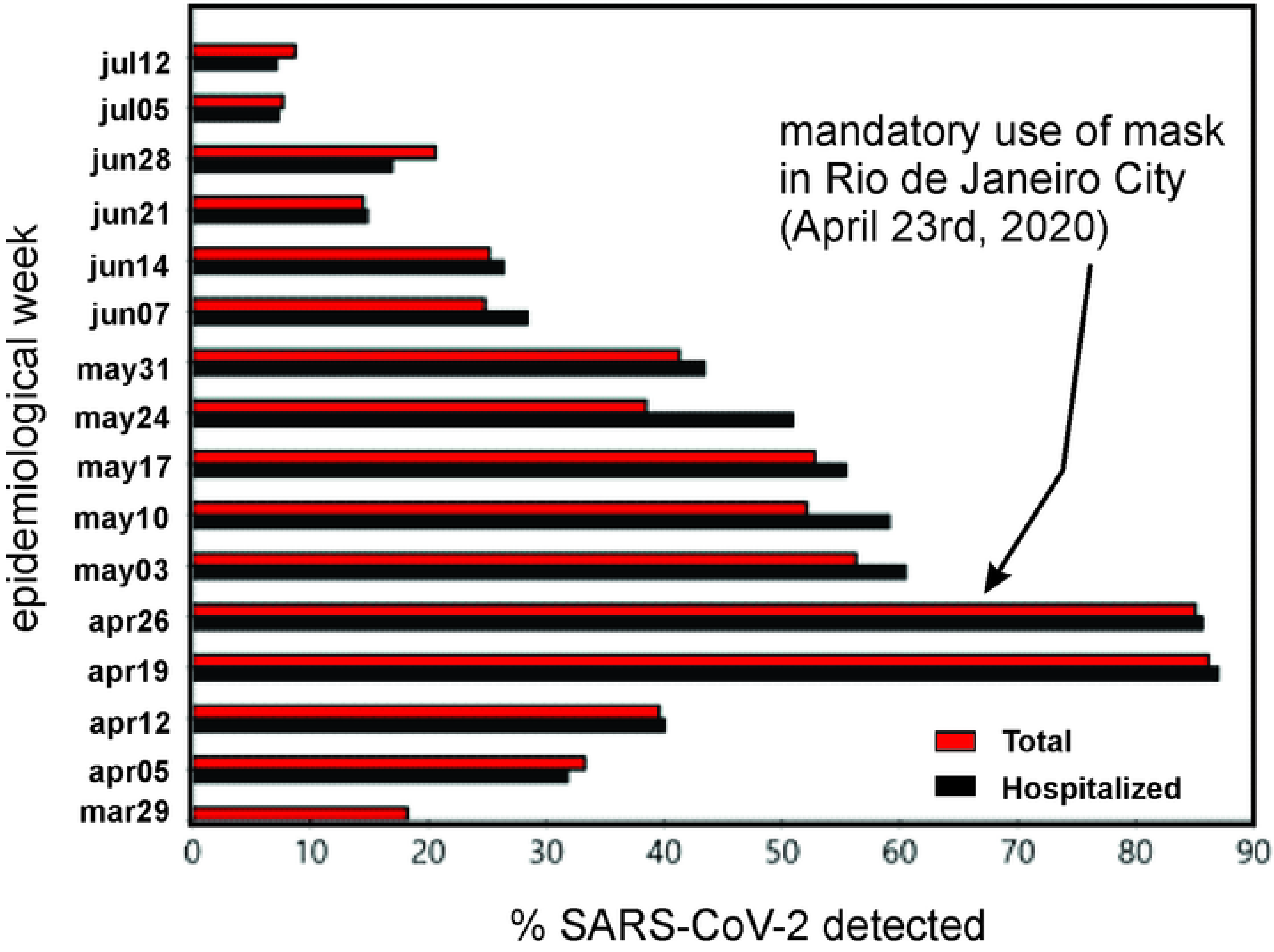

Supplement: Attachment — Submitted filename: PONE-D-20-25540_.pdf [file pone.0245019.s004.pdf]
